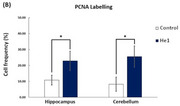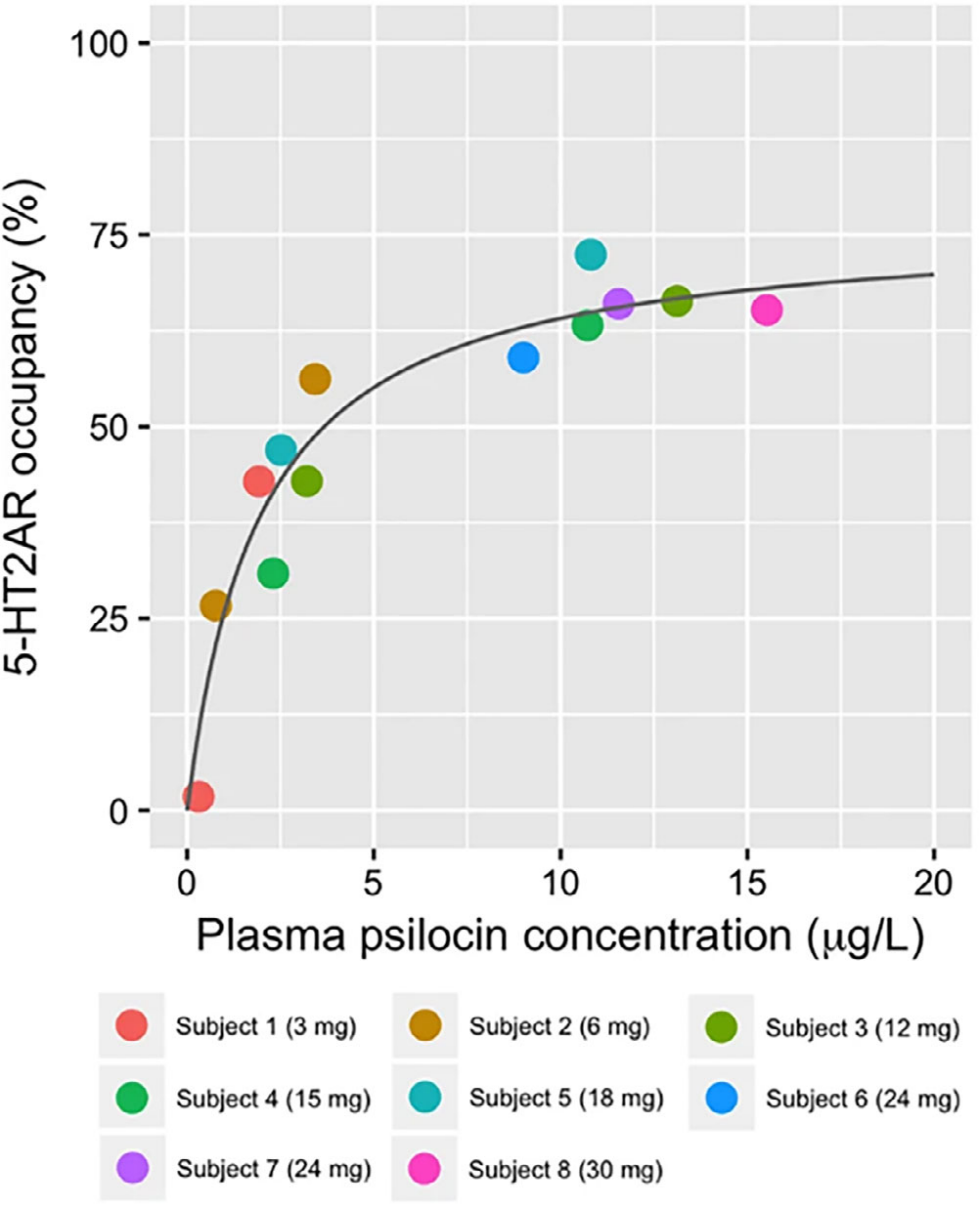# Magic mushrooms‐ *P. cubensis* and *H. erinaceus* a possible novel treatment for Alzheimer's disease symptoms

**DOI:** 10.1002/alz70856_097076

**Published:** 2025-12-24

**Authors:** Anjali Rose Friedman

**Affiliations:** ^1^ Adams State University, Alamosa, CO, USA

## Abstract

**Background:**

Recent studies have highlighted the neuroprotective properties of certain mushrooms. Proposing a research hypothesis for a novel field of study into mitigating the neurological and neuropsychological symptoms associated with Alzheimer's disease (AD) and possible cure with natural methods. *Psilocybe cubensis* and *Hericium erinaceus* have proven to show therapeutic effects for anxiety and depression. *H. erinaceus* chemical extracts have shown to enhance cognitive function, memory and motor skills. Which are notable symptoms of Alzheimer's disease.

**Method:**

Oral supplementation of 1g‐3.5g *P. cubensis*, depending on individual patient whether to increase dosage and if ongoing treatment is needed. Tracking AD patient mental health symptoms prior to experiment and after initial first use, using the Beck Depression Inventory (BDI). As well as using a CT or MRI to check for changes to neuro‐inflammation levels. Next, Continuous oral supplementation of chemical extracts of *H. erinaceus*, known as hericenones and erinacines, which are known to mimic Nerve Growth Factors (NGF) and Brain Derived Neurotropic Factors (BDNF) which can possibly protect the neurons from hyperphosphorylated tau tangles.

**Result:**

Inferring that the use of the *P. cubensis* will increase production of microglial cells and Insulin Degrading Enzymes (IDE) which may break down beta‐amyloid plaques and reduce neuro‐inflammation. Psilocybin also interacts with the 5HT‐2A serotonergic pathway which activity is dampened in AD patients leading to mood disorders. Inferring that *H. erinaceus* extracts will reduce tau and beta‐amyloid levels and reverse degradation of myelin sheath as well as increased cell proliferation in hippocampus.

**Conclusion:**

Suggesting that more novel approaches should be proposed and increased funding and lenience into the tightly restricted experimental use of *P. cubensis* as it has promising potential in the realm of mental and brain health.